# Supplementary material for: Divergent urban storm response to convective, frontal and tropical systems
Source: Nature. 2026 May 20;653(8116):1078–84. doi: 10.1038/s41586-026-10479-7 (PMC13216064; doi:10.1038/s41586-026-10479-7)
Supplement: Supplementary file 1 — This file contains Supplementary Figs. 1–10, Supplementary Tables 1–3 and Supplementary References. [file 41586_2026_10479_MOESM1_ESM.docx]

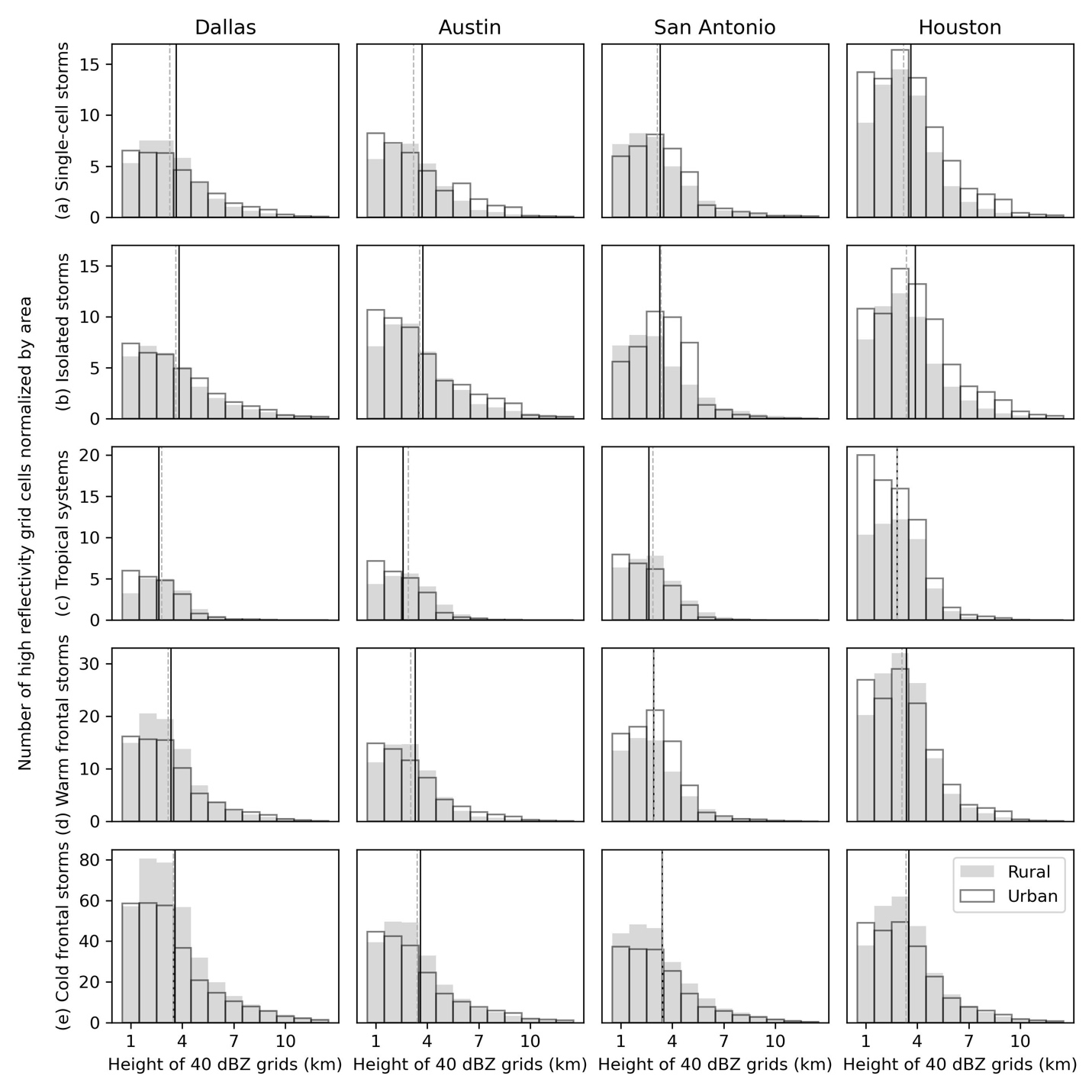


**Fig. S1: Histogram of the height distribution of high reflectivity grid cells (≥40 dBZ) in urban and rural domains.** The rural histogram represents the average result across four rural domains. The majority of these histograms show more high reflectivity grids at 1 km altitude in cities than rural domain, which may be related to uncertainties in low-level radar measurements caused by ground blockage and clutter. Comparing the average height of high reflectivity grid cells (vertical lines) with the lowest 1 km excluded, single-cell storms are consistently and significantly higher in urban areas compared to rural areas across four cities (Paired Wilcoxon signed-rank test: *p* < 0.001, *r* = 0.57, *n* = 92), whereas tropical cyclones are consistently but not significantly lower (*p* = 0.18, *r* = 0.21, *n* = 40). The south domain data over the ocean are excluded for Houston.


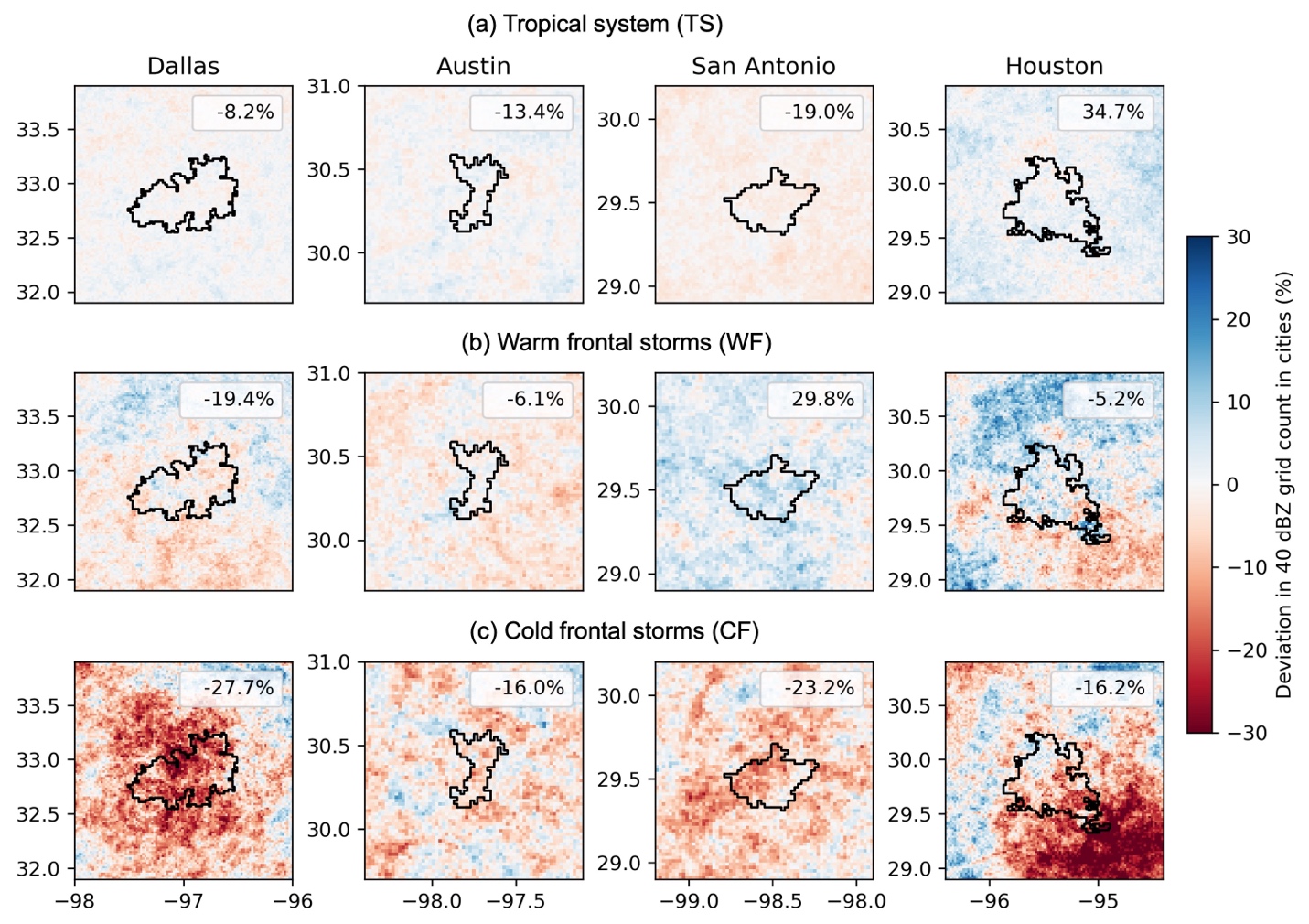


**Fig. S2: Spatial anomalies of high reflectivity grid cells (≥40 dBZ) for different storm types.** Spatial distributions of anomalies associated with (a) tropical system, (b) warm frontal, and (c) cold frontal storms around Texas cities, relative to their rural counterparts, similar to Fig. 3a and Fig. 3b for single-cell storms and isolated storms. The accumulated anomaly number within the city boundary is indicated, which varies above and below zero by chance across different cities for tropical systems and warm frontal storms. However, all four cities exhibit negative anomalies of 40 dBZ grids for urban cold fronts, indicating reduced cold frontal storm intensity around cities.


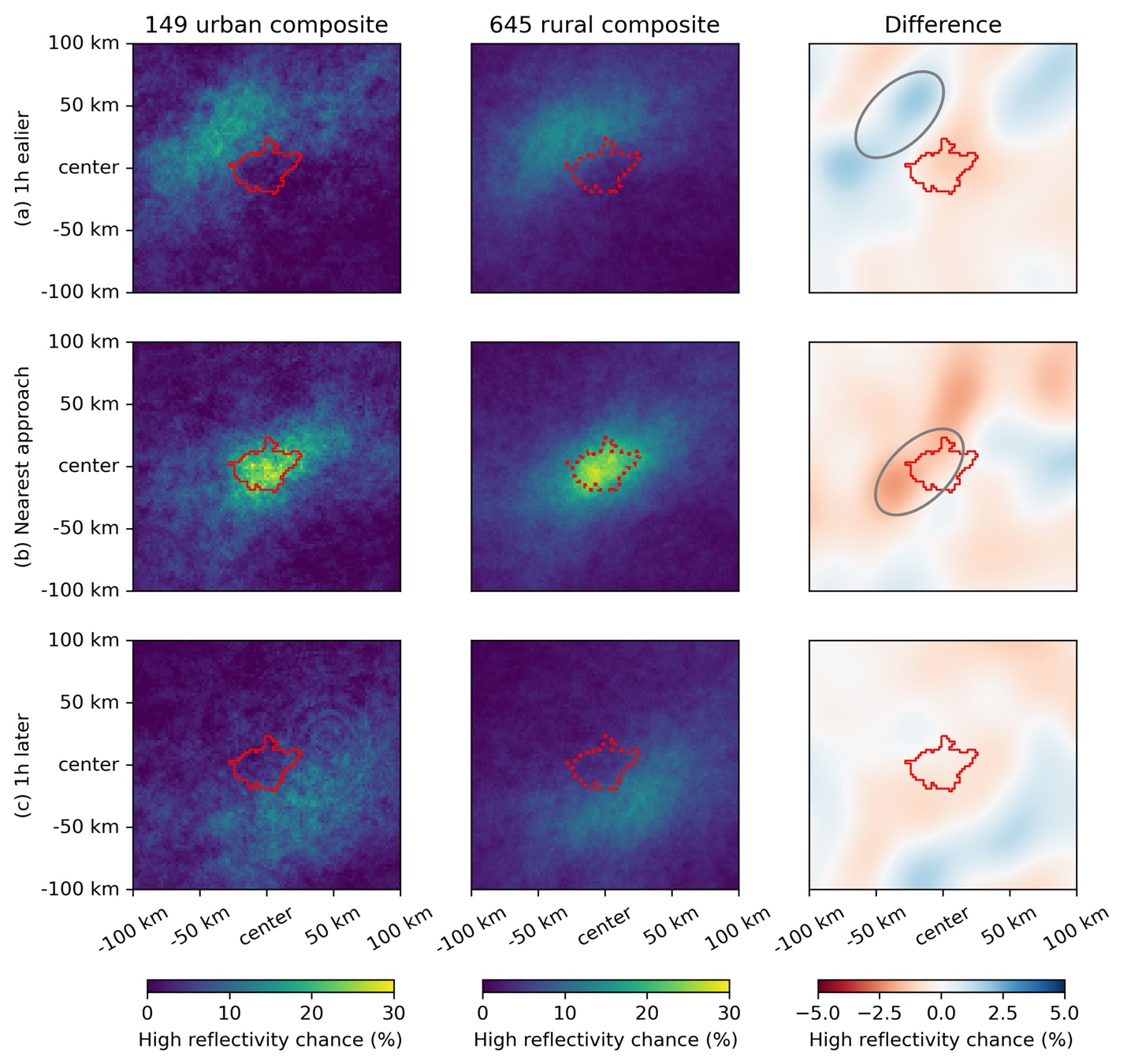


**Fig. S3: Cold frontal storms weaken over San Antonio during passage and show enhancement ahead of arrival.** San Antonio shows a similar cold front modification pattern as Austin. Upon arrival, we found a significant reduction in reflectivity intensity over San Antonio and non-significant increase in high reflectivity grids northwest of the city one hour before.


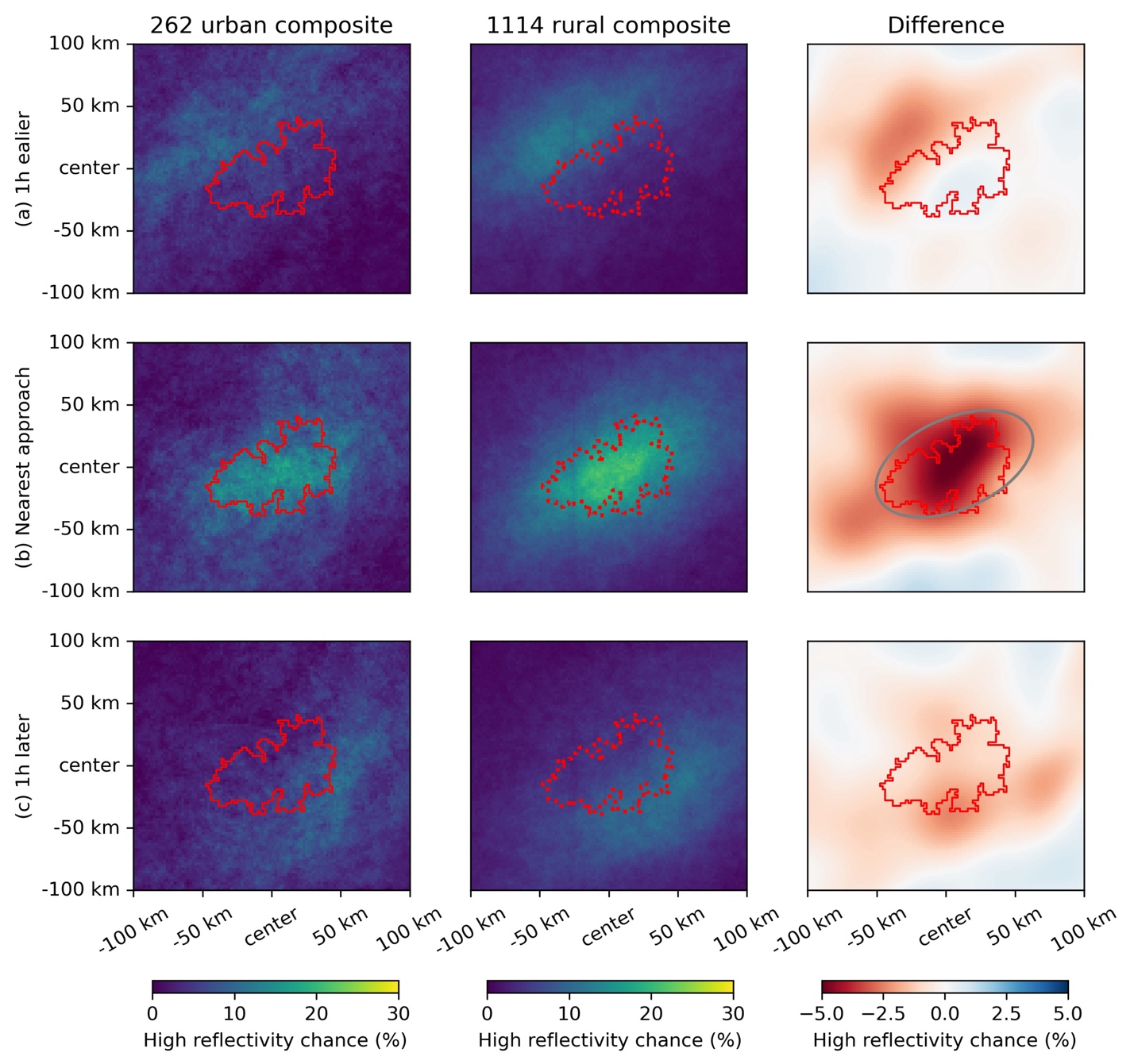


**Fig. S4: Cold frontal storms weaken over Dallas.** With a much larger city footprint than Austin and San Antonio, cold frontal storms show attenuation one hour before reaching the Dallas city center.


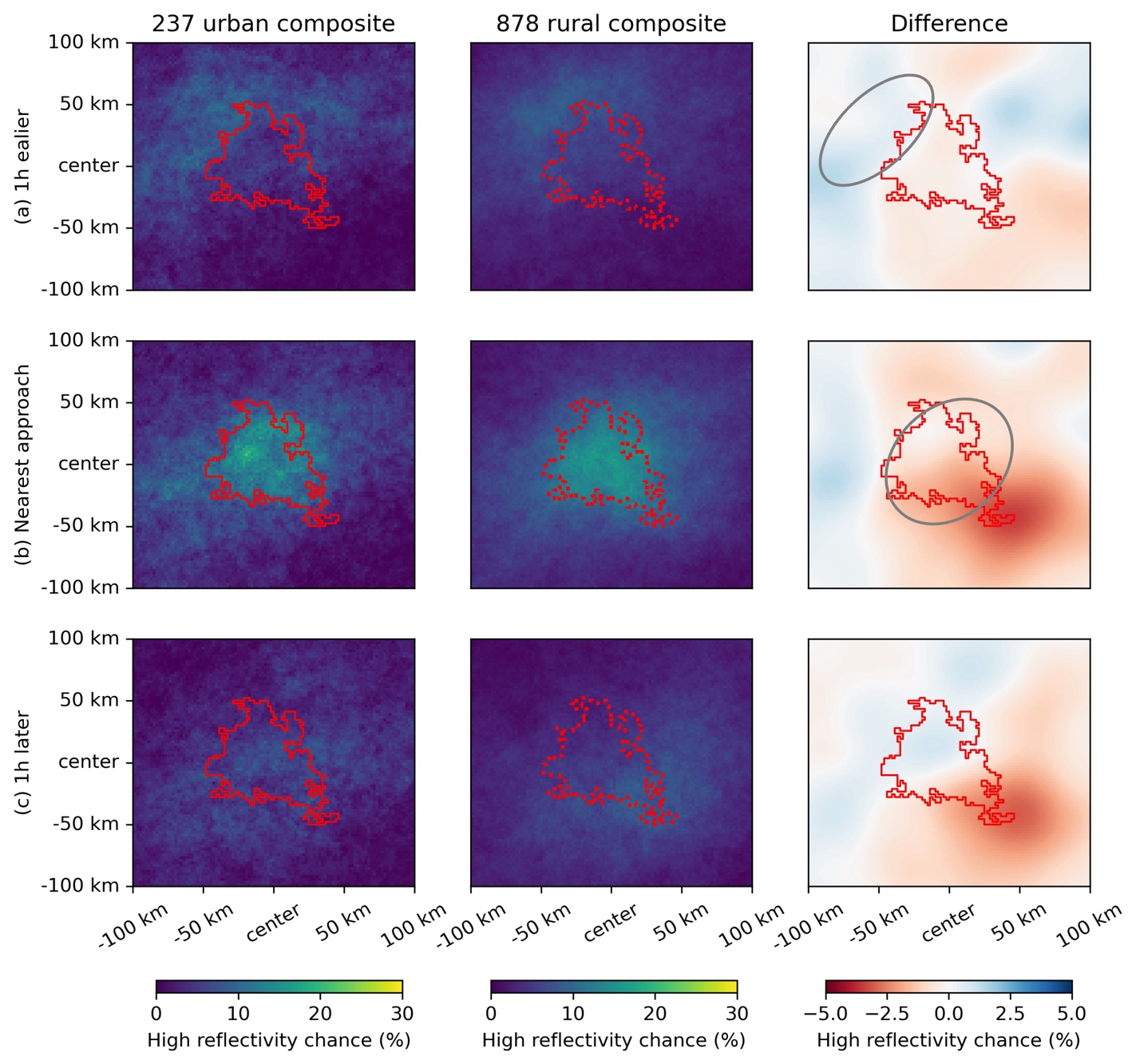


**Fig. S5: Cold frontal storms weaken over Houston and show slight enhancement ahead of arrival.** Due to more southern location of Houston and its proximity to the Gulf Coast, cold fronts weaken greatly before reaching Houston and leave no clear frontal signature in the figure.


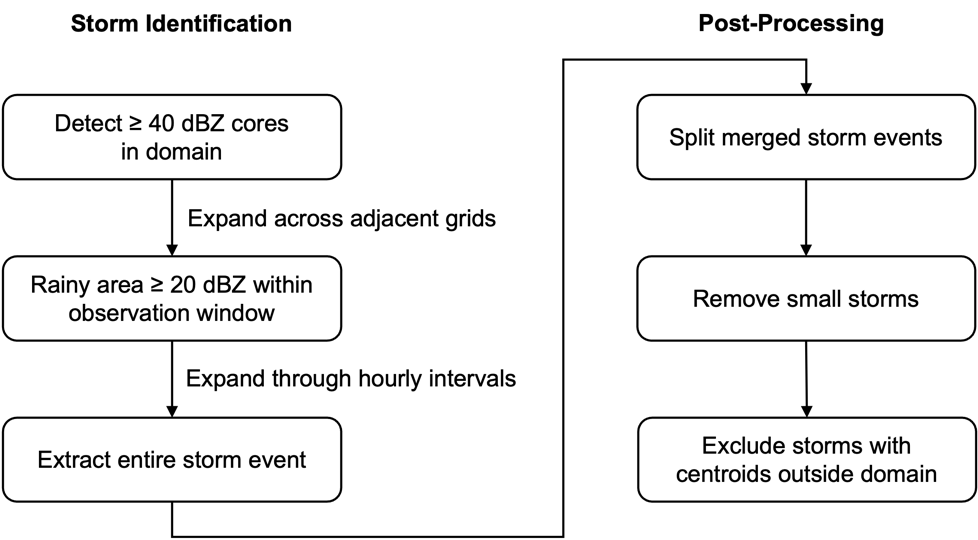


**Fig. S6: Flowchart illustrating storm identification and post-processing procedures.**


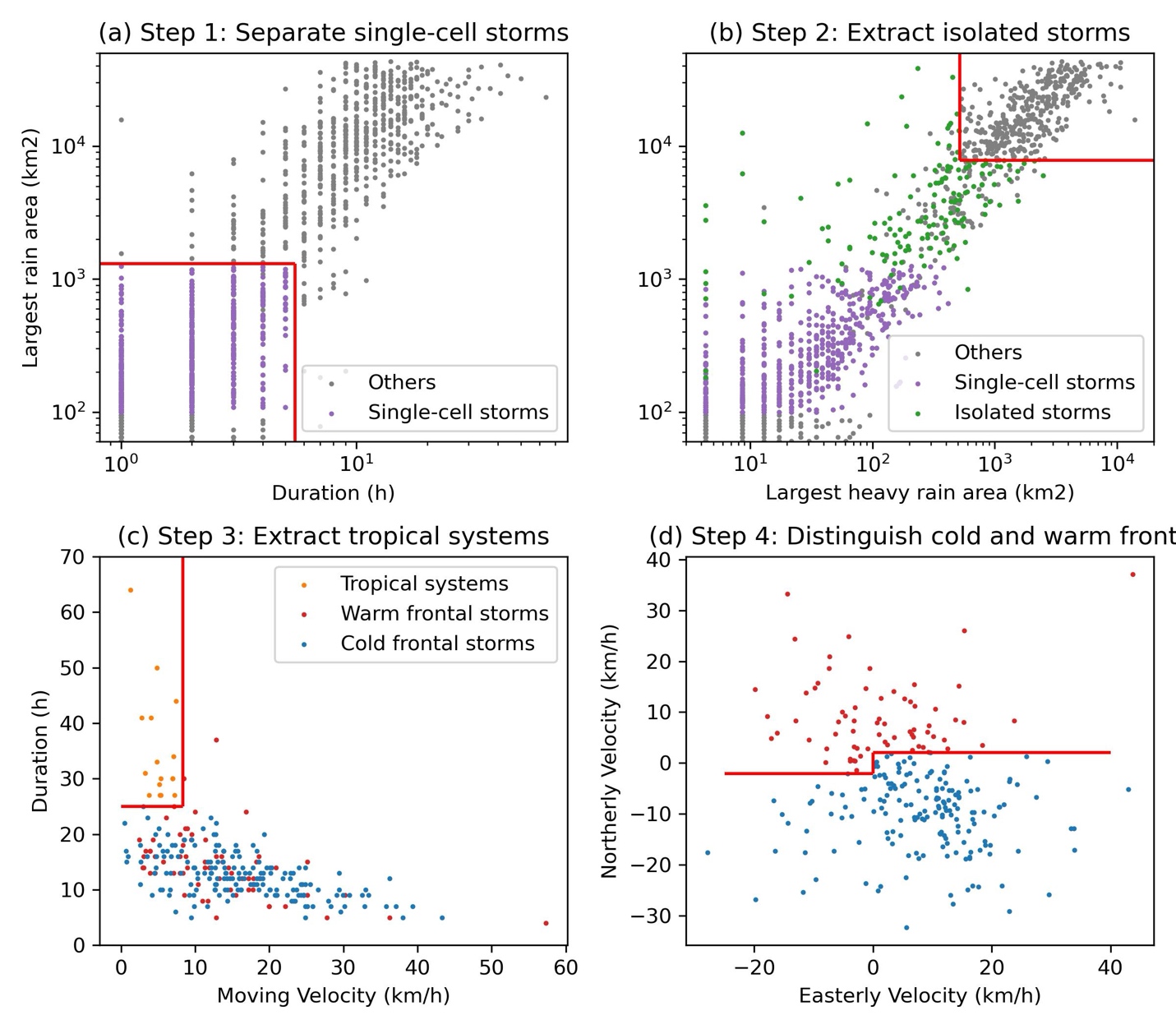


**Fig. S7: Storm classification for 940 storm events detected in Austin across 23 warm seasons.**

**
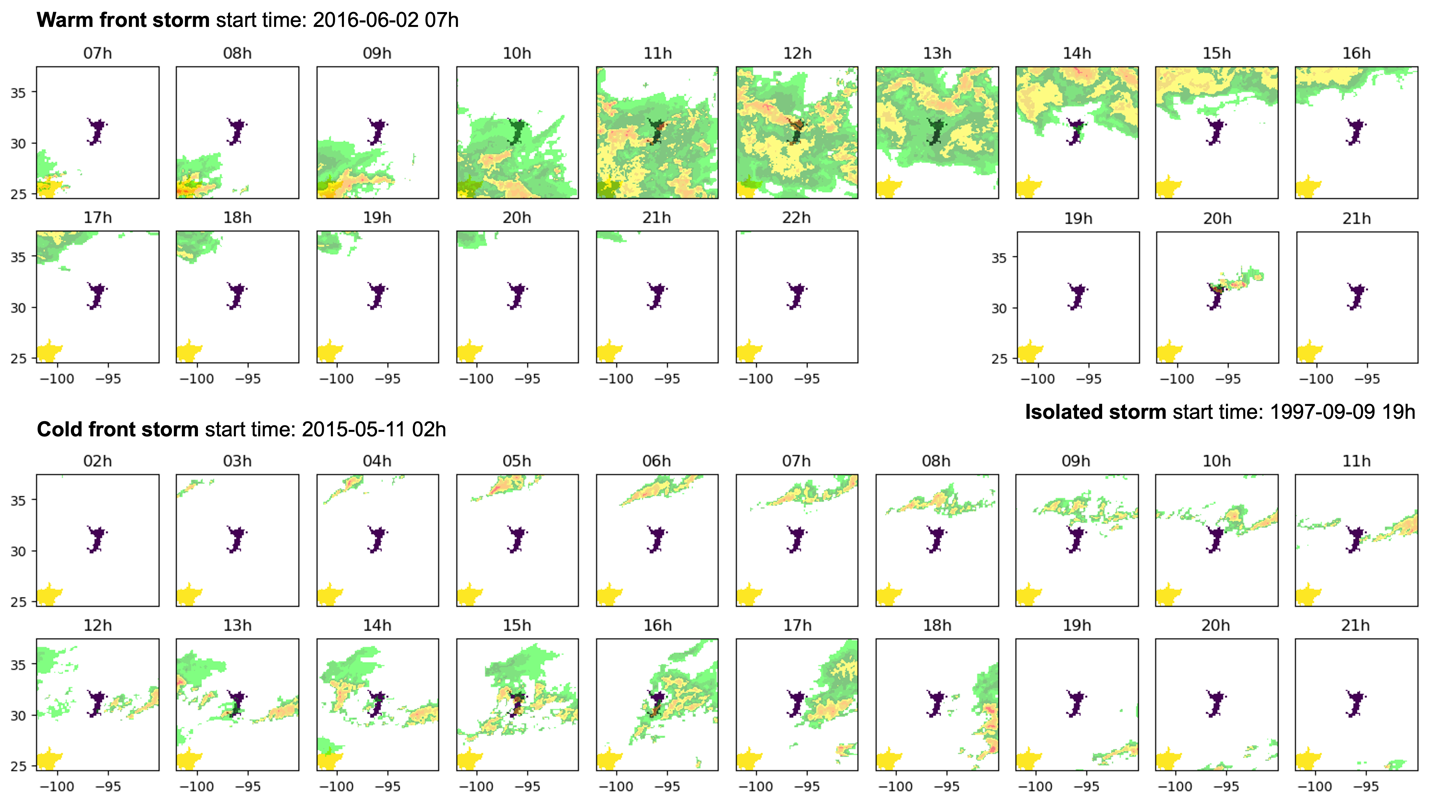
**

**Fig. S8: Hourly snapshots of selected isolated, warm and cold frontal storms cases passing Austin.** Animations are available for download from Zenodo (10.5281/zenodo.15933280)**.**

**
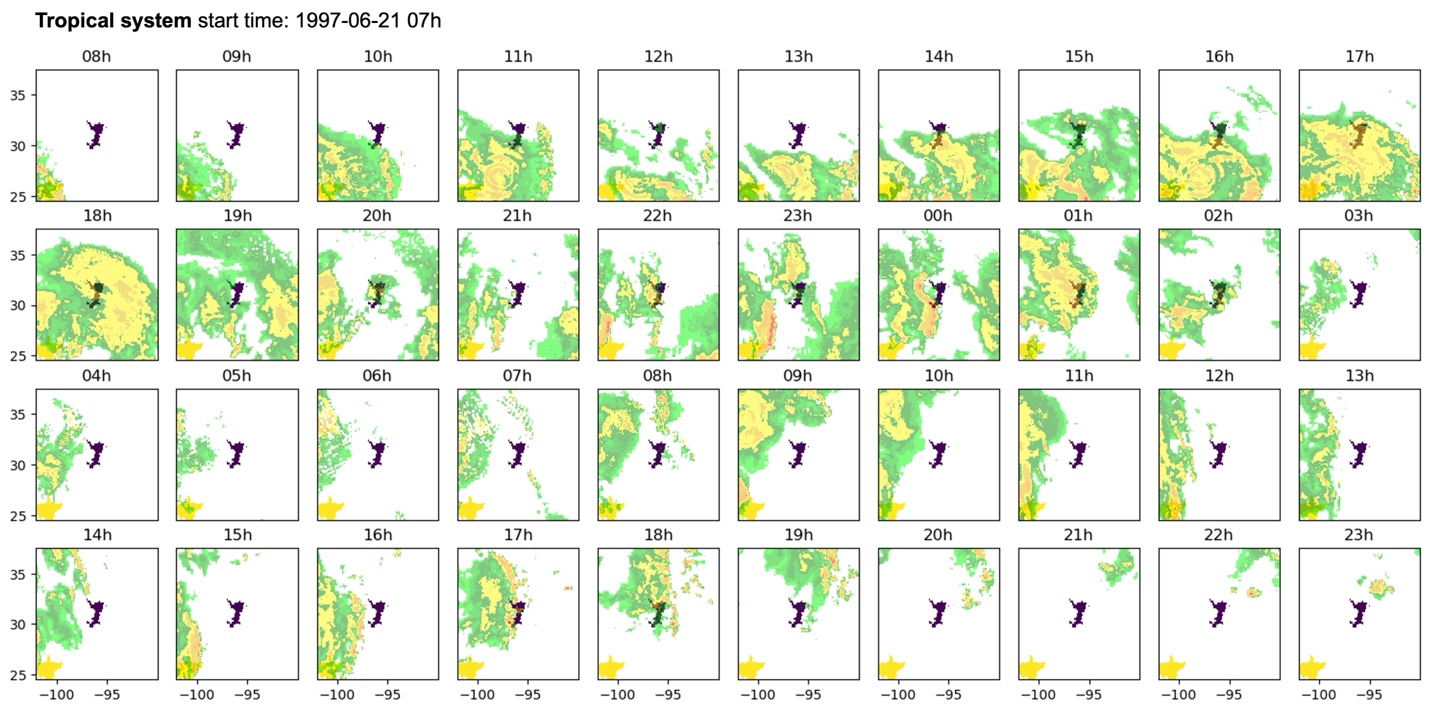
**

**Fig. S9: Hourly snapshots of a selected tropical system case passing Austin.** Animations are available for download from Zenodo (10.5281/zenodo.15933280)**.**

**
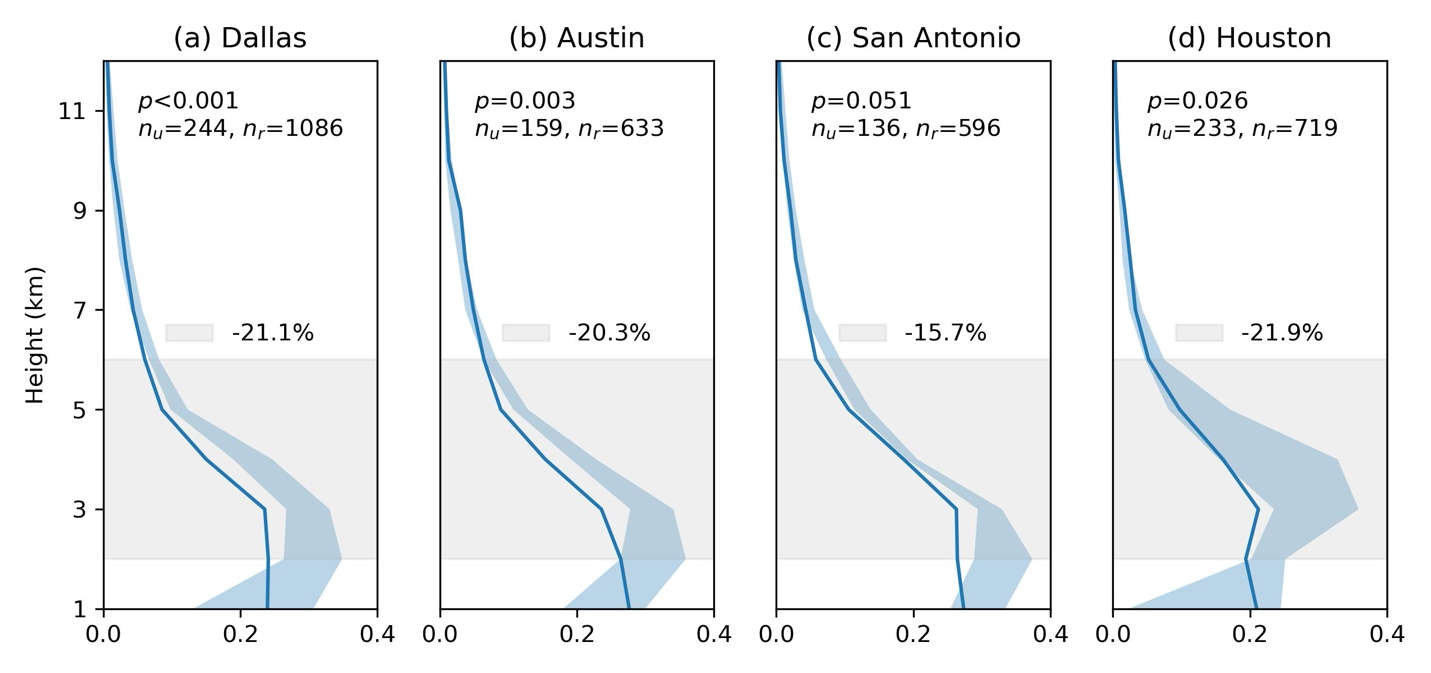
**

**Fig. S10. Sensitivity of cold front intensity comparisons between urban and rural areas.** The decreased intensity remains statistically significant with elevated reflectivity thresholds (25 dBZ for rainfall and 45 dBZ for heavy rainfall).

**Table S1: Paired Wilcoxon signed-rank test results for urban–rural differences in storm numbers.** The null hypothesis (H₀) is that the yearly storm number in urban areas is not greater than the average of their four rural counterparts. 23 years (1995–2017) observations across four cities yield 92 paired data for sufficient statistical power (*n* = 92).

|  | W | *p* value | *r* value |
| --- | --- | --- | --- |
| Single-cell storm | 2673 | 0.0059 | 0.29 |
| Isolated storm | 2691 | 0.0011 | 0.34 |
| Tropical system | 1141.5 | 0.41 | 0.086 |
| Warm front | 1907 | 0.36 | 0.095 |
| Cold front | 1813.5 | 0.60 | 0.055 |

**Table S2. Sensitivity of urban–rural storm number comparisons to reflectivity thresholds.** Similar to Extended Data Table 1 and Table S1, it shows storm counts identified with elevated reflectivity thresholds (25 dBZ for rainfall and 45 dBZ for heavy rainfall) and the significant test. The null hypothesis (H₀) is that the yearly storm number in urban areas is not greater than the average of their four rural counterparts.

|  | Dallas | Austin | San Antonio | Houston | W | *p* value | *r* value |
| --- | --- | --- | --- | --- | --- | --- | --- |
| Single-cell storms | 931 (63.0%) | 268 (43.2%) | 333 (49.9%) | 2609 (74.9%) | 2717 | 0.0068 | 0.28 |
| Isolated storms | 194 (13.1%) | 114 (18.4%) | 109 (16.3%) | 433 (12.4%) | 2665 | 0.0033 | 0.31 |
| Tropical system | 10 (0.7%) | 16 (2.6%) | 9 (1.3%) | 26 (0.7%) | 1123.0 | 0.40 | 0.093 |
| Warm front | 99 (6.7%) | 64 (10.3%) | 81 (12.1%) | 182 (5.2%) | 1994 | 0.23 | 0.12 |
| Cold front | 244 (16.5%) | 159 (25.6%) | 136 (20.4%) | 233 (6.7%) | 1792.5 | 0.75 | 0.033 |
| All | 1478 | 621 | 668 | 3483 |  |  |  |

**Table S3. Verification of 26 tropical influenced weather systems identified in Houston.** The start dates and duration indicate the periods when each event entered the observation window, rather than the full lifecycle of the weather system. In some cases, long-lived hurricanes or tropical storms are separated into two events due to spatial or temporal discontinuities within the observational window.

|  | Start date | Weather event | Duration (h) | Reference |
| --- | --- | --- | --- | --- |
| 1 | 6/28/95 | Tropically influenced convective system | 53 |  |
| 2 | 8/23/96 | Hurricane Dolly | 47 | Tropical Cyclone Reports |
| 3 | 8/28/96 | Hurricane Dolly | 87 | Tropical Cyclone Reports |
| 4 | 7/30/97 | Isolated convective storms | 53 |  |
| 5 | 8/6/97 | Stationary front | 47 | Texas Climate Report |
| 6 | 9/21/97 | Tropically influenced cold frontal storm | 101 | Texas Climate Report |
| 7 | 9/8/98 | Tropical Storm Frances | 127 | Tropical Cyclone Reports |
| 8 | 9/14/98 | Tropical Storm Frances | 71 | Tropical Cyclone Reports |
| 9 | 6/6/01 | Tropical Storm Allison | 54 | Tropical Cyclone Reports |
| 10 | 6/8/01 | Tropical Storm Allison | 50 | Tropical Cyclone Reports |
| 11 | 8/28/01 | Tropical Storm Dean | 138 | Tropical Cyclone Reports |
| 12 | 9/22/01 | Cold Front | 48 | Texas Climate Report |
| 13 | 7/15/02 | Tropical wave | 50 | National Weather Service |
| 14 | 8/14/02 | Tropically influenced convective system | 51 | Texas Climate Report |
| 15 | 9/20/03 | Tropically influenced MCS | 60 |  |
| 16 | 5/13/04 | Tropically influenced MCS | 46 | Curtis et al., 2004 |
| 17 | 6/25/04 | Subtropical jet influenced frontal storm | 65 | Texas Climate Report |
| 18 | 7/3/07 | Tropically enhanced upper-level trough | 66 | Texas Climate Report |
| 19 | 9/10/09 | Hurricane Ike | 94 | Fanelli et al., 2009 |
| 20 | 6/29/10 | Hurricane Alex | 91 | Tropical Cyclone Reports |
| 21 | 9/6/10 | Tropical Storm Hermine | 70 | Tropical Cyclone Reports |
| 22 | 9/19/13 | Hurricane Ingrid | 56 | Tropical Cyclone Reports |
| 23 | 6/16/15 | Tropical Storm Bill | 71 | Tropical Cyclone Reports |
| 24 | 8/13/16 | Tropical easterly wave | 96 | Brown et al., 2020 |
| 25 | 6/21/17 | Tropical Storm Cindy | 66 | Tropical Cyclone Reports |
| 26 | 8/24/17 | Hurricane Harvey | 154 | Tropical Cyclone Reports |

**References**

Brown, V.M., Keim, B.D., Kappel, W.D., Hultstrand, D.M., Peyrefitte Jr, A.G., et al. How rare was the August 2016 South-Central Louisiana heavy rainfall event? *J. Hydrometeorol.* *21*(4), pp.773-790 (2020).

Curtis, L., KWTX-TV, W. & Moller, A.R. A multi-platform analysis of the Central Texas floods of May 13, 2004. In *22nd Conference on Severe Local Storms (2022)*.

Fanelli, C., Fanelli, P., & Fenstermacher, L. E. *Hurricane Ike*. NOAA water level and meteorological data report. National Oceanic and Atmospheric Administration. Silver Spring, MD (2009).

National Hurricane Center. Tropical Cyclone Reports: Atlantic Hurricane Season. National Oceanic and Atmospheric Administration. (n.d.).

Texas A&M University. Texas Climate Bulletin index. Texas Climate. <https://climatexas.tamu.edu/products/texas-climate-bulletins/index.html> (n.d.).
